# Continuous glucose monitoring in critically ill adults: comparison of two different calibration protocols

**DOI:** 10.1186/cc12397

**Published:** 2013-03-19

**Authors:** L Leelarathna, S English, H Thabit, K Caldwell, J Allen, K Kumareswaran, M Wilinska, M Nodale, J Mangat, M Evans, R Burnstein, R Hovorka

**Affiliations:** 1 University of Cambridge, UK; 2 Addenbrookes Hospital, Cambridge, UK

## Introduction

We evaluated the clinical and numerical accuracy of the Freestyle Navigator continuous glucose monitoring (CGM) system, in critically ill adults using two different methods of calibration. Studies looking at intensive glucose control have yielded conflicting results with increased rates of iatrogenic hypoglycemia. Availability of accurate real-time glucose information may improve safety and efficacy of glucose control in the critical care unit.

## Methods

In a randomized prospective trial, paired CGM and reference glucose (hourly arterial blood gas (ABG)) were collected from 24 adults with critical illness (age 60 ± 14 years, BMI 29.6 ± 9.3 kg/m^2^, APACHE score range 6 to 19) and hyperglycemia (glucose ≥10 mmol/l or treated with intravenous insulin), over 48 hours. In 12 subjects, CGM was force-calibrated at variable 1 to 6 hourly intervals using ABG glucose (FC arm). In the other 12 subjects, the sensor was calibrated according to the manufacturer's instructions (1, 2, 10, 24 hours after insertion) using arterial blood and built-in glucometer (MC arm).

## Results

Two groups had similar characteristics at baseline. A total of 1,060 CGM/ABG pairs were analyzed and reference glucose ranged from 4.3 to 18.8 mmol/l. Median (IQR) absolute relative deviation was lower in the FC arm (7.0% (3.5, 13.0) vs. 12.8% (6.3, 21.8), FC vs. MC, *P *0.001). Similarly, the percentage of points in the Clarke error grid zone A points meeting ISO criteria were higher with FC (87.8% vs. 70.2%). Sensor bias (median (IQR)) was significantly lower in the FC arm (-0.1 (-0.7, 0.4) mmol/l vs. -1.1 (-2.3, -0.1) mmol/l, *P *0.001). The median (IQR) interval between calibrations in FC arm was 169 (122, 213) minutes.

## Conclusion

CGM accuracy in the MC arm was comparable with accuracy in subjects with type 1 diabetes. Further significant improvements to CGM accuracy in critical care are possible by increasing the frequency of calibrations. Such accurate CGM may provide valuable information to guide insulin therapy in critically ill subjects.
